# Right sided arcus aorta as a cause of dyspnea and chronic cough

**DOI:** 10.1186/2049-6958-7-37

**Published:** 2012-10-23

**Authors:** Sevket Ozkaya, Bilal Sengul, Semra Hamsici, Serhat Findik, Unal Sahin, Aziz Gumus, Halit Cinarka

**Affiliations:** 1 Rize University, Faculty of Medicine, Department of Pulmonary Medicine, Rize, Turkey; 2 Samsun Chest Diseases and Thoracic Surgery Hospital, Department of Pulmonary Medicine, Samsun, Turkey; 3 Ondokuz Mayis University, Faculty of Medicine, Department of Pulmonary Medicine, Samsun, Turkey

**Keywords:** Asthma, Cough, Dyspnea, Right sided arcus aorta, Spirometry

## Abstract

**Background:**

Right sided arcus aorta (RSAA) is a rare condition that is usually asymptomatic. Patients may present with exertional dyspnea and chronic cough. A recent article suggested that RSAA should be included in the differential diagnosis of asthma, especially in patients with intractable exertional dyspnea. We aimed to present the clinical, radiologic and spirometric features of thirteen patients with RSAA observed in four years at the Rize Education and Research Hospital and Samsun Chest Diseases and Thoracic Surgery Hospital.

**Methods:**

The characteristics of patients with RSAA, including age, gender, symptoms, radiologic and spirometric findings, were retrospectively evaluated.

**Results:**

A total of thirteen patients were diagnosed with RSAA. Their ages ranged from 17 to 86 years and the male to female ratio was 11:2. Seven of the patients (54%) were symptomatic. The most common symptoms were exertional dyspnea, dysphagia and chronic cough. Five patients had received treatment for asthma with bronchodilators. Spirometry showed intrathoracic tracheal obstruction in five patients.

**Conclusions:**

The RSAA anomaly occurs more frequently than might be estimated from the number of patients who are detected. Patients with intractable exertional dyspnea and chronic cough should be evaluated for the RSAA anomaly by thoracic CT.

## Background

Right sided arcus aorta (RSAA) is a rare condition that is usually asymptomatic. A recent article suggested that RSAA should be included in the differential diagnosis of asthma, especially in patients with intractable exertional dyspnea [1]. There are insufficient information and correlations between RSAA anomaly and dyspnea. We aimed to present the clinical, radiologic and spirometric features of thirteen patients with right sided arcus aorta.

## Methods

We previously reported on seven patients with RSAA [1]. We identified six new patients with RSAA at the Rize Education and Research Hospital in 2010. We aimed to present the clinical, radiologic and spirometric features of thirteen patients with RSAA observed in four years at the Rize Education and Research Hospital and Samsun Chest Diseases and Thoracic Surgery Hospital. The institutional review board gave approval and written consent was obtained from the patients for the use of medical records and images. The characteristics of these patients, including age, gender, symptoms, radiologic and spirometric findings, were retrospectively evaluated and are presented in this report. The patient’s consent forms were obtained.

## Results and discussion

**Figure 1:**
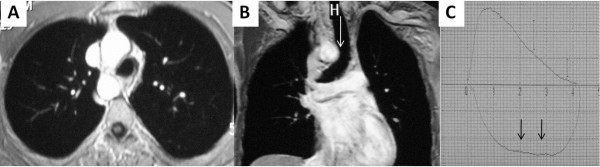
**Thoracic MRI and spirometry images; right sided arcus aorta with tracheal compression as seen on thoracic MRI (1A, 1B).** Spirometry showed a plateau on flow-volume curves throughout inhalation (1**C**).

**Figure 2:**
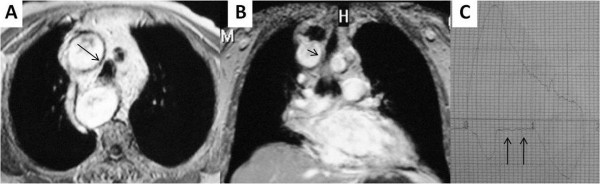
**Thoracic MRI and spirometry images; right sided arcus aorta with tracheal compression as seen on thoracic MRI (2A, 2B, black arrows).** Spirometry showed a plateau on flow-volume curves throughout inhalation (2**C**).

**Figure 3:**
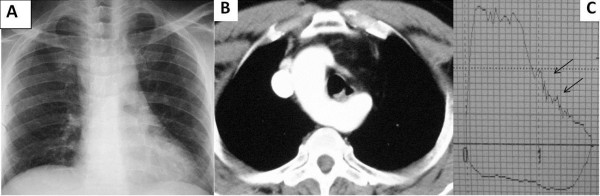
**Thoracic MRI and spirometry images; right sided arcus aorta as seen on thoracic MRI (3A, 3B).** Spirometry showed the “saw-tooth” sign (black arrows) and a plateau on flow-volume curves throughout exhalation and inhalation (3**C**).

**Figure 4:**
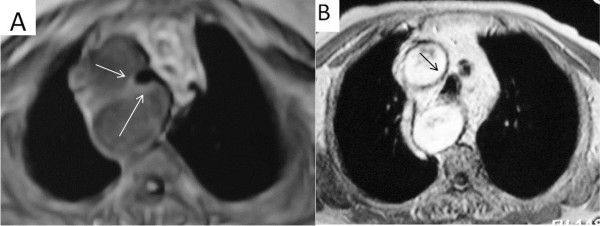
Thoracic MRI and CT images; Thoracic CT and MRI images showing marked compression of the trachea in patients with hypertension and RSAA (arrows).

**Figure 5:**
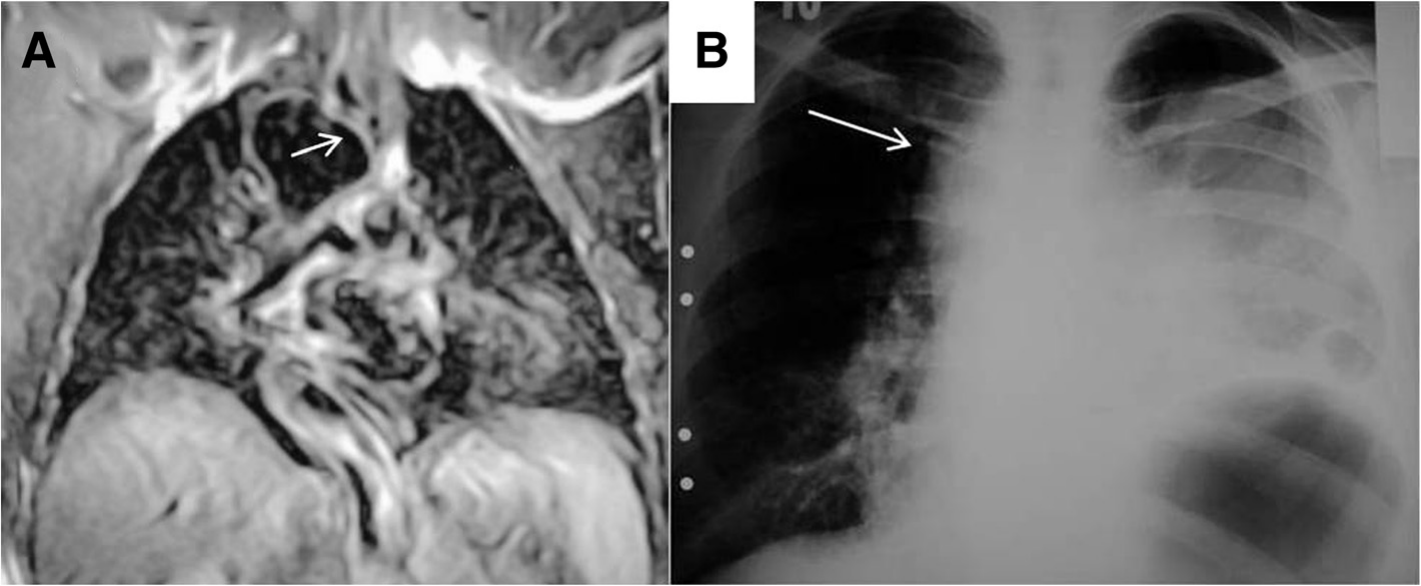
**Thoracic MRI and Chest radiograph images; marked compression of the trachea is seen on thoracic MRI (5A).** A chest radiograph showed a lung cancer in the left hemithorax and right sided arcus aorta (5**B**, white arrow).

The characteristics of the patients are presented in Table 1. Patients age ranged from 17 to 86 years and the male to female ratio was 11:2. Seven patients (54%) were symptomatic due to external compression of the trachea as a result of RSAA (Figures 1, 2, 3, 4 and 5). The most common symptoms were exertional dyspnea, dysphagia and chronic cough. Five patients (38%) were misdiagnosed as having asthma due to the symptoms of exertional dyspnea. Three patients (23%) were using long-acting beta agonists (LABA) and inhaled corticosteroids, although there was no improvement in their symptoms.

**Table 1:** Characteristics of patients

**Patients**	**Age/Gender**	**Symptoms**	**Spirometry**	**Causes of symptoms**
**1.**	17 y/Male	Exertional dyspnea	Intrathoracic airway obstruction	Tracheal compression from RSAA
**2.**	55 y/Male	Cough Dyspnea	Restriction	Lung Cancer
**3.**	44 y/Male	Dyspnea Dysphagia	Normal	Tracheal and esophageal compression from RSAA
**4.**	54 y/Female	Dyspnea Dysphagia	Restriction	Tracheal and esophageal compression from RSAA and Obesity
**5.**	52y/Male	Dyspnea	Obstruction	COPD
**6.**	35 y/Male	Chest pain	Normal	Non-specific upper airway infection
**7.**	27y/Male	Exertional dyspnea	Intrathoracic airway obstruction	Tracheal compression from RSAA
**8.**	53y/Male	Cough Dyspnea	Intrathoracic airway obstruction	Tracheal compression from RSAA
**9.**	86y/Male	Dyspnea Dysphagia	Intrathoracic airway obstruction	Tracheal and esophageal compression from RSAA
**10.**	48y/Male	Cough	Intrathoracic airway obstruction with “saw-tooth” sign	Tracheal compression from RSAA
**11.**	27y/Male	Cough	Normal	Non-specific upper airway infection
**12.**	60y/Female	Cough	Normal	Sinusitis
**13.**	25y/Male	Cough Dyspnea	Small airways obstruction	Bronchiolitis and sinusitis

**Figure 6:**
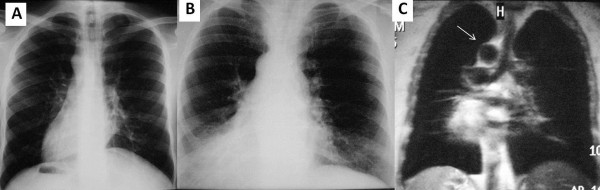
**Thoracic MRI and Chest radiograph images Chest radiographs showing dextrocardia with RSAA (6A, 6B).** Thoracic MRI showed that there was no tracheal compression due to RSAA (6**C**, white arrow).

In the other six patients symptoms, when present, were related to lung cancer, COPD, bronchiolitis with Kartagener’s syndrome, and non-specific upper airway infection. Chest X-ray images showed the absence of the arcus aorta shadow on the left side of the mediastinum (Figure 3A). Two patients had RSAA with Kartagener’s syndrome (situs inversus totalis and dextrocardia) and RSAA due to dextrocardia (Figure 6). There was no tracheal compression in patients with RSAA and Kartagener’s syndrome. Two patients had hypertension. Exertional dyspnea was more pronounced in patients with hypertension and RSAA (Figure 4). The spirometric flow-volume curves during expiration and inspirations showed intrathoracic tracheal obstruction in five patients, and one of them had the “saw-tooth” sign (Figures 1C, 2C and 3C). Other spirometric findings were reported as restrictive in two patients and obstructive in two patients. Spirometry was normal in four patients. The diagnosis of RSAA was confirmed by thoracic CT and/or MRI in all patients. Thoracic MRI also revealed marked narrowing of the tracheal air column due to external compression by the RSAA in five patients (Figures 1B, 2B, 4 and 5A).

Right aortic arches are a diverse set of anomalies including the second and third most common vascular rings. Because the aorta usually arises to the right of the midline (irrespective of arch sidedness) and, with one exception, right aortic arches originate where aorta begin descending on the right, there is a chance that the right mainstem bronchus will be compressed between the sagittally oriented ascending and descending aorta as described above. In *situssolitus* (normal arrangement of asymmetrical body organs and atria) the descending aorta is left sided at the diaphragm irrespective of arch sidedness. Thus with right aortic arches the aorta must go from being right-sided at the level of the bronchi to left-sided at the diaphragm. In most cases this is a gradual diagonal passage with no perceptible indentation on the esophagus. However, in one situation – right aortic arch with left descending aorta – similar to left arch right descending above, the crossing is abrupt and associated with a ring [2]. It was reported to occur in 0.1 - 0.2% of adults [3]. Assman, first described the roentgenographic studies of this anomaly and Renander described the first case reported in the English literature [4]. We previously reported the incidence of RSAA as 0.16% [1]. The RSAA anomaly is usually asymptomatic. However, in the present study, 54% of the patients were symptomatic due to this defect. According to previous reports, some patients present with dysphagia and dyspnea on exertion [1,4-7], while others receive a clinical diagnosis of exercise-induced asthma. In the present study, seven patients were symptomatic due to external compression of the trachea and esophagus as a result of RSAA. The RSAA anomaly may be more common in the community than estimated from the number of patients who are detected.

Spirometry may be helpful for diagnosing suspected tracheal compression in symptomatic patients [7]. The expiratory loop of spirometry flow-volume curves showed flattening in two patients. In addition, the inspiratory loop of flow-volume curves showed flattening in two patients. Flattening of flow-volume curve during in- and expiration was observed in one patient. This variation may be due to the level at which tracheal compression occurs. If tracheal compression occurs in the upper part of the trachea, flattening of the flow-volume curve occurs during expiration, as observed in two patients. However, if tracheal compression occurs in the lower part of the trachea, flattening of the flow-volume curve occurs during inspiration, as observed in two other patients (Figures 1B, 1C). One patient demonstrated the “saw-tooth” sign on the flow-volume curve. In this patient, the RSAA and aberrant left brachiocephalic artery surrounded the trachea, and may have affected the flow-volume curves due to arterial pulsation (Figure 3B). There was greater exertional dyspnea in the two patients with hypertension and RSAA, due to marked compression of the trachea by the RSAA (Figure 4). Hypertension may contribute to increased dyspnea in patients with RSAA.

IIf there is no shadow of arcus aorta on the left side of the mediastinum on chest radiography, RSAA should be suspected. Thoracic CT and MRI are the best methods to diagnose RSAA, and MRI should be the preferred one. MRI is a non-invasive diagnostic tool and can clearly show the relationships between intrathoracic vascular structures and the trachea. In the present study external tracheal compression due to RSAA was demonstrated by thoracic MRI. RSAA should be included in the differential diagnosis of asthma, especially in patients with intractable exertional dyspnea [1].

## Conclusions

The RSAA anomaly is more common than might be estimated from the number of patients who are detected. Spirometric findings may help in the identification of tracheal compression due to RSAA. All patients with intractable exertional dyspnea should be evaluated for the RSAA anomaly by thoracic CT.

## Consent

Written informed consent was obtained from the patients for publication of this manuscript and any accompanying images. A copy of the written consent is available for review by the Editor-in-Chief of this journal.

## Abbreviations

RSAA: Right Sided Arcus Aorta.

## Competing interest

The authors declare that they have no competing interests.

## Authors’ contributions

SO, BS, SH, SF, US, AG and HC have made substantial contributions to conception and design, or acquisition of data, or analysis and interpretation of data. All authors read and approved the final manuscript.
